# Myocardial injury and clinical outcome in octogenarians after non–ST-elevation myocardial infarction

**DOI:** 10.3389/fcvm.2024.1422878

**Published:** 2024-07-22

**Authors:** Toni Pätz, Thomas Stiermaier, Moritz Meusel, Iris Reinhard, Philipp-Johannes Jensch, Elias Rawish, Juan Wang, Hans-Josef Feistritzer, Andreas Schuster, Alexander Koschalka, Torben Lange, Johannes T. Kowallick, Steffen Desch, Holger Thiele, Ingo Eitel

**Affiliations:** ^1^ Department of Cardiology, Angiology and Intensive Care Medicine, German Center for Cardiovascular Research (DZHK), University Heart Center Lübeck, Medical Clinic II, University of Lübeck, Lübeck, Germany; ^2^ Department of Biostatistics, Central Institute of Mental Health, Medical Faculty Mannheim/Heidelberg University, Mannheim, Germany; ^3^ The Second People’s Hospital of Yibin, Yibin, Sichuan, China; ^4^ Department of Internal Medicine/Cardiology and Leipzig Heart Institute, Heart Center Leipzig at University of Leipzig, Leipzig, Germany; ^5^ Department of Cardiology and Pneumology, University Medical Center Göttingen, Georg-August University, Germany and German Centre for Cardiovascular Research (DZHK), Partner Site Lower Saxony, Göttingen, Germany; ^6^ Institute for Diagnostic and Interventional Radiology, German Center for Cardiovascular Research (DZHK), University Medical Center Göttingen, Georg-August University, Göttingen, Germany

**Keywords:** elderly, myocardial infarction, cardiac imaging, outcome, MACE

## Abstract

**Introduction:**

The aim of this study was to analyze age-associated myocardial injury and clinical outcome after non-ST-elevation myocardial infarction (NSTEMI).

**Methods:**

This prospective, multicenter study consists of 440 patients with NSTEMI enrolled at 7 centers. All patients were treated with primary percutaneous coronary intervention and underwent cardiac magnetic resonance (CMR) imaging 1–10 days after study inclusion. CMR parameters of myocardial injury and clinical outcome were evaluated by creating 2 subgroups: <80 years vs. ≥80 years. The clinical endpoint was the 1-year incidence of major adverse cardiac events (MACE) consisting of death, re-infarction and new congestive heart failure.

**Results:**

Elderly patients ≥80 years accounted for 13.9% of the study population and showed a divergent cardiovascular risk profile compared to the subgroup of patients <80 years. CMR imaging did not reveal significant differences regarding infarct size, microvascular obstruction, left ventricular ejection fraction or multidimensional strain analysis between the study groups. At 1-year follow-up, MACE rate was significantly increased in patients ≥80 years compared to patients aged <80 years (19.7% vs. 9.6%; *p* = 0.019). In a multiple stepwise logistic regression model, the number of diseased vessels, aldosterone antagonist use and left ventricular global longitudinal strain were identified as independent predictors for MACE in all patients, while there was no independent predictive value of age regarding 1-year clinical outcome.

**Conclusion:**

This prospective, multicenter analysis shows that structural and functional myocardial damage is similar in younger and older patients with NSTEMI. Furthermore, in this heterogeneous but also clinically representative cohort with reduced sample size, age was not independently associated with 1-year clinical outcome, despite an increased event rate in patients ≥80 years.

## Introduction

In an aging society, people ≥80 years represent an increasing proportion of patients (1) and physicians will be faced with patients who have a high burden of disease (2), especially cardiovascular diseases including myocardial infarction (3). Since elderly patient cohorts are underrepresented in published literature (4, 5), it is important to consider these patients in clinical studies to increase our knowledge and ensure evidence-based treatment also in this patient subgroup. In this regard, acute myocardial infarction is of particular interest. Despite of all improvement in medical treatment, mortality rate in patients with myocardial infarction is still stagnating on a high level (6) with a significant proportion of death in elderly patients within the first year after non-ST-elevation myocardial infarction (NSTEMI) (7). Hence, there is still a need for research to identify predictors for major adverse cardiac events (MACE) after myocardial infarction especially in elderly high-risk patients. It is also unclear whether the myocardial damage differs between the elderly and younger NSTEMI patients or not. For example, study results indicate a different collateral formation depending on patient's age (8), which strengthens the theory of potential different myocardial damage in elderly patients compared to their younger counterparts. Moreover, higher myocardial vulnerability in elderly patients could be caused by several other pre-existing age-related differences, like reduced beta-adrenergic sympathetic responsiveness (9). It had been discussed that this decreased inotropic beta-adrenergic responsiveness may affect cardiovascular function in the post-infarction period by limiting contractile reserve in the region remote from the infarct, which led to a reduced ability of that myocardium to compensate for the contractile performance lost as the result of the infarct itself (10). In addition, a higher diastolic LV pressure in elderly patients (11) and an increased LV stiffness (12) may impair coronary perfusion and contribute to adverse outcomes after infarction in older patients (10). To evaluate the prognosis after myocardial infarction, left ventricular ejection fraction, as an established and validated method to reflect myocardial damage, is not helpful to detect discrete changes in cardiac function and showed to have some major limitations in patients with survived myocardial infarction (13). Cardiac magnetic resonance (CMR) imaging provides valuable insights into the structural damage following myocardial infarction and modern CMR-based assessment of myocardial deformation with the feature tracking (FT) technique emerged as superior tool to estimate myocardial function (14). Previous studies emphasized the prognostic implications of these CMR parameters for post-infarction risk stratification (15–18). However, none of these studies focused solely on patients with NSTEMI. Due to the lack of evidence in CMR-FT in NSTEMI and age-related differences in CMR-based infarction characteristics in elderly patients further research is needed. The aim of this study was therefore to compare the extent of post-infarction myocardial damage assessed with CMR imaging and the risk of adverse clinical events in younger and older patients with NSTEMI.

## Materials and methods

### Study population and design

This study is a sub-analysis of a previously published NSTEMI trial (Thrombus Aspiration in Thrombus Containing Culprit Lesions in Non–ST-Elevation; TATORT-NSTEMI trial) (19). The detailed study design has already been published elsewhere (20). Briefly, the TATORT-NSTEMI trial was designed as a prospective, controlled, multicenter, randomized, open-label trial comparing percutaneous coronary intervention (PCI) with routine thrombus aspiration with PCI without thrombus aspiration in NSTEMI patients. A total of 440 patients were included at seven participating sites, 221 of these patients were randomized to aspiration thrombectomy and 219 patients to standard PCI. Afterwards, all patients were examined using CMR imaging. The main inclusion criteria were ischemic symptoms lasting >20 min; occurrence of last symptoms <72 h before randomization; cardiac troponin T levels above the 99th percentile; culprit lesion containing thrombus [Thrombolysis In Myocardial Infarction (TIMI) thrombus grades 2–5 within the lesion]; and intended early PCI. Patients with cardiogenic shock, STEMI, no identifiable culprit lesion, coronary morphology ineligible for thrombectomy (e.g., very tortuous vessels, severe calcification); indication for acute bypass surgery; age younger than 18 and older than 90 years; contraindications to treatment with heparin, aspirin, or thienopyridines; pregnancy; current participation in another clinical study; comorbidity with a limited life expectancy <6 months; and contraindications to CMR at study entry were excluded (19). Overall, aspiration thrombectomy in conjunction with PCI in NSTEMI with a thrombus-containing lesion did not lead to a reduction in microvascular obstruction (19). The TATORT-NSTEMI trial is registered with ClinicalTrials.gov (NCT01612312). All patients gave written informed consent before randomization. The study was approved by all local ethical committees of the participating sites.

### CMR imaging protocol

On days 1–10, all patients were examined at all participating sites using the same CMR imaging protocol on clinical 1.5 or 3.0 Tesla MR scanners (19–22). The detailed scan protocol has been described previously (19, 20). In brief, left ventricular (LV) volumes and function were assessed with standard steady-state free precession technique, T2-weighted triple-inversion recovery turbo spin-echo images were obtained for determination of edema/myocardium at risk, and T1-weighted inversion recovery turbo gradient-echo sequences approximately 15 min after intravenous administration of gadolinium-based contrast agent (late gadolinium enhancement images) were used to assess infarct size and microvascular obstruction. The images of the CMR scan were sent on secure media to CMR core laboratories for assessment by fully blinded operators.

### CMR analysis

All CMR parameters were assessed as described previously using certified evaluation software (cmr42, Circle Cardiovascular Imaging Inc., Calgary, Alberta, Canada and 2D CPA MR, Cardiac Performance Analysis, Version 1.1.2, TomTec Imaging Systems, Unterschleissheim, Germany) (19). Regions of infarcted myocardium and microvascular obstruction were delineated with a semi-automated computer-aided threshold detection (>5 standard deviations of remote myocardium in ≥10 adjacent myocardial pixels) and expressed as the percentage of LV mass (%LV). “Area at risk” was defined as regions with simultaneous myocardial oedema*.* If present, microvascular obstruction was included in the overall infarct size and also quantified separately. LV ejection fraction (EF) was calculated in continuous short axis slices covering the whole ventricle. In addition, CMR-FT was performed in an experienced core laboratory at the University Medical Center Goettingen (23, 24). A semi-automatic process was used, in which LV endocardial borders were manually traced at end-diastole in electrocardiography-gated balanced steady-state free precession short- and long-axis sequences using a point-and-click approach. Afterwards, the software's automatic border tracking algorithm was deployed, which tracks image features throughout the whole cardiac cycle. In addition, tracking accuracy was visually reviewed; if necessary, adjustments were made to the initial contour only. The average of 3 independent repeated measurements in the 4-chamber view was chosen as the final value and images that did not allow for a reliable tracking were excluded from the analysis. Global radial strain (GRS) and global circumferential strain (GCS) were computed as average values from respective short axis slice analyses. Global longitudinal strain (GLS) was defined using average strain curves from 2- and 4-chamber orientations.

### Clinical outcome variables

The detailed outcome definitions of the TATORT-NSTEMI trial with consideration of the clinical outcome committee and the hierarchy of the combined endpoint to avoid double counting of events have already been published elsewhere (19, 20). MACE was defined as a composite of all-cause death, reinfarction, and new congestive heart failure within 1 year after infarction. In order to evaluate the MACE rate depending on the patient's age, we created 2 frequently used and clinically relevant subgroups (2, 25–27): <80 years (*n* = 379, 86.1%) and ≥80 years (*n* = 61, 13.9%).

### Statistical analysis

The statistical analysis was performed with IBM SPSS Statistics 25.0. A two-sided *p*-value of 0.05 and below was classified as statistically significant. Categorical variables were investigated using Chi-square test or Fisher's exact test and are expressed as numbers and percentages. Continuous variables were analyzed using Mann-Whitney-*U*-test and are expressed as median and interquartile range (IQR). Differences with respect to survival functions (time to death) between the two different age groups were tested by means of the log-rank test. Kaplan-Meier curves illustrate the survival rates graphically.

Clinical characteristics, CMR parameters, and clinical outcome were compared between NSTEMI patients <80 years and ≥80 years. Influencing determinants of MACE were analyzed using logistic regression analysis. All results of these logistic regressions are presented as odds ratios (OR) and 95% confidence intervals (CI). Moreover, in order to analyze the independent predictive value of significant parameters in the first step, we used a stepwise forward regression to assess the impact of these parameters on the incidence of MACE at 1-year follow-up in a multiple binary regression model. In this regression model, age was included as a continuous variable. For all regression analyzes, an odds ratio >1 means that with the presence of the characteristic (binary data) or with increasing value of the variable (continuous data), the probability of MACE increases. For missing data no imputation was performed.

## Results

### General results and age-associated outcome

A total of 440 patients with a median age of 68.5 years [interquartile range (IQR) 58–75] had been included in this study. Table 1 shows the detailed baseline and procedural characteristics of the 2 subgroups. Patients aged ≥80 years (*n* = 61) were less frequently male (*p* < 0.001), had a higher prevalence of hypertension (*p* = 0.007) and diabetes mellitus (*p* = 0.005), were less frequently smokers (*p* < 0.001) and had a lower body mass index (*p* = 0.001). Moreover, more patients ≥80 years had been assigned into a higher Killip class on admission compared to patients <80 years (*p* = 0.018). The left anterior descending artery and the left circumflex artery were more frequently affected in older patients, while younger patients presented more right coronary artery culprit lesions (*p* = 0.017).

**Table 1:** Baseline characteristics.

Variable	All patients (*n* = 440)	<80 years (*n* = 379)	≥80 years (*n* = 61)	*p*
Age (years)	68.5 (58, 75)	66 (55, 72)	83 (81, 86)	* *
Male sex	322/440 (73.2)	290/379 (76.5)	32/61 (52.5)	**<0**.**001**
Cardiovascular risk factors				
Current Smoking	159/419 (37.9)	152/358 (42.5)	7/61 (11.5)	**<0.001**
Hypertension	346/439 (78.8)	290/378 (76.7)	56/61 (91.8)	** **
Hyperlipoproteinemia	166/439 (37.8)	146/378 (38.6)	20/61 (32.8)	**0.007**
Diabetes mellitus	128/439 (29.2)	101/378 (26.7)	27/61 (44.3)	0.383
Body mass index (kg/m^2^)	27.76 (25.01, 30.71)	28.03 (25.50, 30.88)	25.17 (23.82, 28.94)	**0.005** **0.001**
Previous myocardial infarction	42/439 (9.6)	36/378 (9.5)	6/61 (9.8)	1.000
Previous PCI	41/439 (9.3)	37/378 (9.8)	4/61 (6.6)	0.421
Previous CABG	17/439 (3.9)	14/378 (3.7)	3/61 (4.9)	0.648
Systolic blood pressure, mmHg	143 (123, 159)	142 (122, 158)	144.5 (126.25, 160)	0.107
Diastolic blood pressure, mmHg	80 (70, 90)	80 (70, 90)	80 (69.25, 90)	0.575
Heart rate, beats/min	76 (67, 86)	76 (67, 86)	75 (67, 84)	0.556
Killip class on admission				**0**.**018**
1	389/440 (88.4)	341/379 (90)	48/61 (78.7)	
2	44/440 (10)	33/379 (8.7)	11/61 (18)	
3	6/440 (1.4)	5/379 (1.3)	1/61 (1.6)	
4	1/440 (0.2)	0/379 (0)	1/61 (1.6)	
Number of diseased vessels				0.770
1	192/440 (43.6)	168/379 (44.3)	24/61 (39.3)	
2	148/440 (33.6)	126/379 (33.2)	22/61 (36.1)	
3	100/440 (22.7)	85/379 (22.4)	15/61 (24.6)	
Infarct related artery		* *	*** ***	**0**.**017**
Left anterior descending	151/440 (34.3)	125/379 (33.0)	26/61 (42.6)	** **
Left circumflex	173/440 (39.3)	148/379 (39.1)	25/61 (41.0)	
Left main	1/440 (0.2)	0/379 (0)	1/61 (1.6)	
Right coronary artery	105/440 (23.9)	98/379 (25.9)	7/61 (11.5)	
Bypass graft	10/440 (2.3)	8/379 (2.1)	2/61 (3.3)	
TIMI flow grade before PCI				0.354
0	167/440 (38)	146/379 (38.5)	21/61 (34.4)	
1	34/440 (7.7)	32/379 (8.4)	2/61 (3.3)	
2	128/440 (29.1)	106/379 (28.0)	22/61 (36.1)	
3	111/440 (25.2)	95/379 (25.1)	16/61 (26.2)	
Stent implanted	421/440 (95.7)	365/379 (96.3)	56/61 (91.8)	0.162
TIMI flow grade post PCI				0.150
0	13/440 (3)	9/379 (2.4)	4/61 (6.6)	
1	5/440 (1.1)	5/379 (1.3)	0/61 (0)	
2	32/440 (7.3)	30/379 (7.9)	2/61 (3.3)	
3	390/440 (88.6)	335/379 (88.4)	55/61 (90.2)	
Concomitant medications				
Glycoprotein IIb/IIIa inhibitor	27/439 (6.2)	24/378 (6.3)	3/61 (4.9)	1.000
Aspirin	436/440 (99.1)	376/379 (99.2)	60/61 (98.4)	0.451
Clopidogrel/prasugrel/ticagrelor	440/440 (100)	379/379 (100)	61/61 (100)	1.000
Beta-blocker	414/440 (94.1)	358/379 (94.5)	56/61 (91.8)	0.385
ACE inhibitor/AT-1 antagonist	373/440 (84.8)	324/379 (85.5)	49/61 (80.3)	0.336
Aldosterone antagonist	77/440 (17.5)	64/379 (16.9)	13/61 (21.3)	0.467
Statin	423/440 (96.1)	365/379 (96.3)	58/61 (95.1)	0.717

Data presented as n/N (%) or median (IQR). *P*-values were calculated for the comparison between patients <80 years and ≥80 years.

Numbers in bold indicate statistical significance.

CABG, coronary artery bypass graft; PCI, percutaneous coronary intervention; TIMI, thrombolysis In Myocardial Infarction, ACE, angiotensin-converting enzyme; AT, angiotensin.

CMR-derived infarct characteristics according to age subgroups are shown in Table 2. CMR imaging was performed in median 3 (IQR 2–4) days after NSTEMI. There was no significant difference between age groups concerning cardiac morphology or function. The only differences were found in endsystolic (*p* < 0.001) and enddiastolic volumes (*p* = 0.014) with a smaller volume in patients aged ≥80 years.

**Table 2:** Cardiac magnetic resonance imaging results.

Variable	All patients	<80 years	≥0 years	*p*
Area at risk (%LV)^a^	20.35 (15.08, 25.33)	20.3 (15.4, 25.1)	21 (13.9, 25.8)	0.743
Infarct size (% LV)^b^	6.3 (2.2, 12.8)	7.0 (2.1, 13.2)	4.6 (2.25, 8.85)	0.081
Microvascular obstruction (% LV)^c^	1.7 (0.7, 3.15)	1.7 (0.7, 3.1)	2.5 (0.2, 17.48)	0.829
LV ejection fraction (%)^d^	50.9 (43.9, 57.38)	50.95 (43.98, 57.48)	49.45 (43.58, 56.5)	0.617
LV end-diastolic volume (ml)^d^	137.75 (113.25, 166.23)	140.75 (116.0, 171.38)	116.3 (92.55, 140.5)	**<0**.**001**
LV end-systolic volume (ml)^d^	65.4 (51.3, 87.7)	66 (52.63, 90.6)	59.5 (41.55, 78.43)	**0**.**014**
Global circumferential strain (%)^e^	−24.72 (−19.16, −29.85)	−24.85 (−19.24, −29.26)	−22.88 (−18.25, −30.97)	0.828
Global radial strain (%)^e^	19.45 (14.61−24.97)	19.6 (14.86, 24.99)	18.64 (11.78, 24.87)	0.187
Global longitudinal strain (%)^f^	−16.77 (−13.09, −19.83)	−16.85 (−13.32, −19.74)	−16.07 (−11.92, −21.25)	0.789

Data presented as median (IQR). *P*-values were calculated for the comparison between patients <80 years and ≥80 years.

Numbers in bold indicate statistical significance.

LV, left ventricular, % LV, percentage of left ventricular mass. a: *n* = 298, b: *n* = 323, c: *n* = 98, d: *n* = 356, e: *n* = 321, f: *n* = 345.

**Figure 1:**
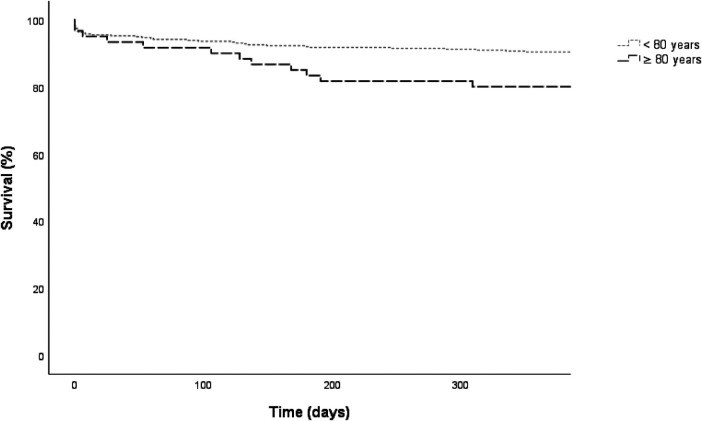
Kaplan-meier survival curves for major adverse cardiac event (MACE) at 1-year follow-up according to age group classification. <80 years: *n* = 376, MACE: *n* = 36 (9.6%), ≥80 years: *n* = 61, MACE: *n* = 12 (19.7%), Log rank *p* = 0.019.

In addition, the incidence of MACE was evaluated according to age categories and showed a significant difference between age groups (*p* = 0.019). At 1-year follow-up, MACE occurred in 36 (9.5%) patients <80 years (death: *n* = 16 (44.4%); reinfarction: *n* = 7 (19.4%); readmission for congestive heart failure: *n* = 13 (36.1%) and in 12 (19.7%) patients ≥80 years (death: *n* = 8 (66.7%); reinfarction: *n* = 2 (16.7%); readmission for congestive heart failure: *n* = 2 (16.7%), Figure 1).

### Predictors of MACE

Tables 3, 4 present the results of the binary logistic regression analyses at 1-year follow-up. In simple logistic regression analysis of all baseline characteristics age (*p* = 0.001), diabetes mellitus (*p* = 0.003), previous myocardial infarction (*p* = 0.018), Killip class on admission (*p* < 0.001), number of diseased vessels (*p* = 0.013), coronary arterial bypass grafting (*p* = 0.008) as the infarct related artery, stent implanted (*p* = 0.037), aspirin (*p* = 0.035), beta-blocker (*p* < 0.001), angiotensin converting enzyme (ACE) inhibitor/(angiotensin) AT-1 antagonist (*p* = 0.046), aldosterone antagonist (*p* = 0.029) and statins (*p* = 0.003) showed significant predictive associations with MACE. In addition, LV EF (*p* = 0.005), LV endsystolic volume (*p* = 0.015), global circumferential strain (*p* = 0.023), global radial strain (*p* = 0.006) and GLS (*p* < 0.001) proved to be significantly associated with MACE. After multiple analysis including all significant parameters of simple binary regression, number of diseased vessels [odds ratio: 1.992 (1.168–3.396), *p* = 0.011], aldosterone antagonist treatment [odds ratio: 2.804 (1.115–7.051), *p* = 0.028] and GLS [odds ratio: 1.102 (1.019–1.193), *p* = 0.015] proved to be significant predictors for MACE at 1-year follow-up (Table 5). Age had no independent impact on the MACE rate at one year follow-up, neither as a binary nor as a continuous variable.

**Table 3:** Binary logistic regression analysis of baseline characteristics and their association with MACE at 1-year follow-up.

Variable	Binary logistic regression MACE OR (95% CI)	*p*
Age (years)	1.051 (1.021–1.082)	**0**.**001**
Age ≥80 years	2.313 (1.127–4.746)	**0**.**022**
Male sex	0.982 (0.500–1.930)	0.959
Cardiovascular risk factors		
Current Smoking	0.835 (0.441–1.581)	0.579
Hypertension	1.403 (0.633–3.110)	0.405
Hyperlipoproteinemia	1.333 (0.727–2.444)	0.353
Diabetes mellitus	2.512 (1.366–4.620)	**0.003**
Body mass index (kg/m^2^)	1.058 (0.996–1.123)	0.066
Previous myocardial infarction	2.658 (1.179–5.988)	**0**.**018**
Previous PCI	1.899 (0.788–4.576)	0.153
Previous CABG	2.622 (0.819–8.393)	0.104
Systolic blood pressure, mmHg	0.996 (0.983–1.009)	0.531
Diastolic blood pressure, mmHg	0.990 (0.969–1.011)	0.348
Heart rate, beats/min	1.013 (0.995–1.032)	0.147
Killip class on admission	3.049 (1.745–5.324)	**<0.001**
Number of diseased vessels	1.614 (1.108–2.352)	**0**.**013**
Infarct related artery		
Left anterior descending	1.276 (0.689–2.360)	0.438
Left circumflex	0.925 (0.498–1.718)	0.806
Left main	-	-
Right coronary artery	0.424 (0.175–1.028)	0.058
Bypass graft	5.803 (1.577–21.358)	**0.008**
TIMI flow grade before PCI	1.112 (0.869–1.421)	0.398
Stent implanted	0.321 (0.110–0.935)	**0**.**037**
TIMI flow grade post PCI	0.730 (0.488–1.090)	0.124
Concomitant medications		
Glycoprotein IIb/IIIa inhibitor	2.497 (0.954–6.532)	0.062
Aspirin	0.119 (0.016–0.864)	**0.035**
Clopidogrel/prasugrel/ticagrelor	-	-
Beta-blocker	0.198 (0.083–0.474)	**<0.001**
ACE inhibitor/AT-1 antagonist	0.484 (0.237–0.987)	0.046
Aldosterone antagonist	2.131 (1.081–4.199)	**0.029**
Statin	0.204 (0.072–0.579)	**0.003**

Numbers in bold indicate statistical significance.

CABG, coronary artery bypass graft; PCI, percutaneous coronary intervention; TIMI, thrombolysis in myocardial infarction; OR, odds ratio; CI, confidence interval; MACE, major adverse cardiac event; ACE, angiotensin-converting enzyme; AT, angiotensin.

**Table 4:** Binary logistic regression analysis cardiac magnetic resonance imaging results and their association with MACE at 1-year follow-up.

Variable	Binary logistic regression MACE OR (CI)	*p*
Area at risk (%LV)	1.030 (0.977–1.086)	0.267
Infarct size (% LV)	1.021 (0.971–1.074)	0.422
Microvascular obstruction (% LV)	1.048 (0.884–1.242)	0.592
LV ejection fraction (%)	0.947 (0.911–0.983)	**0**.**005**
LV end-diastolic volume (ml)	1.006 (0.997–1.014)	0.173
LV end-systolic volume (ml)	1.012 (1.002–1.021)	**0**.**015**
Global circumferential strain (%)	1.066 (1.009–1.126)	**0**.**023**
Global radial strain (%)	0.910 (0.851–0.974)	**0**.**006**
Global longitudinal strain (%)	1.146 (1.064–1.235)	**<0**.**001**

Numbers in bold indicate statistical significance.

OR, odds ratio; CI, confidence interval; LV, left ventricular; % LV, percentage of left ventricular mass; MACE, major adverse cardiac event.

**Table 5:** Multiple stepwise logistic regression analyses of baseline characteristics and cardiac magnetic resonance imaging results and their association with MACE at 1-year follow-up.

Variable	Multiple stepwise binary logistic regression MACE OR (CI)	*p*
Number of diseased vessels	1.992 (1.168–3.396)	**0**.**011**
Aldosterone antagonist	2.804 (1.115–7.051)	**0**.**028**
Global longitudinal strain (%)	1.102 (1.019–1.193)	**0**.**015**

Numbers in bold indicate statistical significance.

OR, odds ratio; CI, confidence interval; MACE, major adverse cardiac event.

## Discussion

This prospective, multicenter study shows no significant differences in CMR imaging derived infarct characteristics in NSTEMI patients aged ≥80 years compared to patients <80 years. Moreover, in our study cohort with a limited sample size of elderly patients age was not independently associated with 1-year clinical outcome despite a numerically increased event rate in elderly patients. By considering the total study population, we were also able to show that aldosterone antagonist treatment, number of diseased vessels and GLS independently predict clinical outcome 1-year after the index event. Additionally, the prognostic value of myocardial strain analysis using CMR was found to be a relevant tool for prediction of clinical events within the first year after NSTEMI. In the context of a heterogeneous but also clinically relevant patient population, these data provide further evidence for the importance of CMR in the elderly, who are currently under-represented in clinical trials (4, 5).

Due to the changing demographics (1) caused by improved public health, nutrition, and medical care and at the same time longer life expectancy (28), elderly people will represent an increasing subset of patients in everyday clinical practice. In addition, octogenarians are one of the fastest growing parts of the population (29). This seems to be elementary since many of daily decisions are based on guidelines in which trial data derived from younger study populations were used to formulate medical advices. On the other hand, complication rates and adverse drug events remain high among older patients (30–32). Currently, different reports addressed these problems in elderly patients and conclude that a paradigm shift in the sense of geriatric cardiology should be of great importance in future practice (29, 33). With this large predefined analysis of a NSTEMI multicenter trial we support this trend by focusing on elderly patients. Participants aged ≥80 years were more likely to be female, weighed less than their younger counterparts, suffered more from hypertension and diabetes mellitus, were less likely smokers, and presented with a higher Killip class on hospital admission compared to patients <80 years, which has already been seen in other study reports (5, 34, 35). It can therefore be assumed that the population shown in our study constitutes a heterogeneous but also representative cohort, which will further improve the transferability of our CMR-derived data to other study populations and thus to the general public.

The evaluation of differences in the extent of myocardial damage by age category was one of the major aspects of our trial. Whether age disparities in clinical care and death after NSTEMI are still present in the current PCI era remain a matter of constant debate and have important clinical implications. Indeed, there is conflicting evidence regarding age-based differences in myocardial injury in patients after myocardial infarction and especially systematic data in NSTEMI patients are lacking. While some study data in patients with STEMI indicate that age has no influence on final infarct size (36), other results from basic cardiovascular research have shown an increased apoptosis of cardiomyocytes in aging hearts (37), which leads to the assumption of greater myocardial damage in older patients. In contrast, an increased formation of coronary collaterals due to the longer history of a pre-existing coronary heart disease was assumed in elderly patients, which could protect from ischemic injury. However, study results also suggest that the prevalence of collaterals decreases with age, which in turn strengthens the hypothesis of a higher myocardial damage in older patients (8). Our data show that there is no significant difference between structural and functional myocardial damage between younger and older patients with NSTEMI. Old patients should therefore offer similar infarct care as our data underline that the efficacy of primary PCI in patients with NSTEMI is not age-dependent.

In an advanced medical development, further parameters beyond the basic risk factors are needed for risk stratification. CMR provides an innovative method to comprehensively examine patients after myocardial infarction. However, little is currently known about the difference in infarct characteristics in older patients compared to younger patients. LV function is a frequently used parameter to estimate the prognosis after acute myocardial infarction (38, 39). Due to the missing differences in CMR-derived LV ejection fraction, this parameter was ineffective to predict MACE rates in our cohort. Overall, there were only few differences in the CMR parameters between age groups. Our data therefore suggest that the unfavorable clinical outcome in older NSTEMI patients is not related to differences in myocardial damage, but rather due to differences in baseline risk and comorbidities. For example, it is known that an increased Killip classification is associated with an increased mortality rate (34). This increased mortality risk can already be observed from Killip class II onwards. In our study, older patients also showed a higher Killip class at admission compared to younger participants, which may have led to the significant difference in the rate of serious cardiac events within the first year after NSTEMI.

This study was primarily designed to evaluate the extent of post-infarction myocardial damage assessed with CMR imaging and the risk of adverse clinical events in younger and older patients with NSTEMI. Age had no independent impact on the MACE rate at one year follow-up, neither as a binary nor as a continuous variable. Since other studies have already shown that age is independently associated with one year outcome after myocardial infarction (40, 41), we assume that our missing differences between age groups are driven by the small sample size of octogenarians*.* In addition, the categorization into specific age cohorts resulted in significant differences in baseline characteristics that were not observed in the original TATORT NSTEMI study. It cannot be excluded that this may have influenced the results of the predictor analysis at one year follow-up. Due to the missing statistical differences*,* we decided to investigate the additive value of the CMR considering the entire cohort. Three parameters proved to predict MACE at 1-year follow-up. First of all, an increasing number of diseased coronary vessels showed a significant association with an increased incidence of MACE within 1 year. This relationship has already been seen in other study results (42) and can be interpreted as the severity of cardiovascular disease*.* Moreover, our study results also suggest that aldosterone antagonist treatment is significantly associated with an increased rate of clinical events at 1-year follow-up. These results do not seem to be unexpected, especially since aldosterone antagonists were initiated in accordance with the guidelines in patients with a LV-EF ≤ 40% (43) to reduce morbidity and mortality in patients after myocardial infarction (44). Nevertheless, it is also known that these patients have significantly increased mortality rates compared to patients with preserved LV function after myocardial infarction (45). In addition, an alternative explanation could be, that the drug therapy was started due to a (resistant) hypertension. The number of patients with hypertension in our cohort was 78.8%. Since a preserved LV EF of approximately 51% was documented on CMR, the high rate of patients on aldosterone antagonist therapy seems to be due to the hypertension therapy. This is also supported by the trend towards hypertensive blood pressure values >140 mmHg in the entire cohort. However, the reason for administration remains speculative, as this aspect was not part of the primary TATORT-NSTEMI trial. It is known that these patients also show clinical events more frequently than patients without hypertension (46). Due to the increased association with serious cardiac events, patients with the need for aldosterone antagonist treatment should be given greater priority in clinical routine, especially in the follow-up care after myocardial infarction.

Finally, GLS, which also proved to be a significant predictor for MACE, represents a possible additional option for risk evaluation. Due to limitations of LV-EF alone for predicting outcome after myocardial infarction (13), additional parameters are required and CMR-FT offers a modern method for outcome prediction in patients with myocardial infarction, but little is known about the parameters in patients with NSTEMI. Study results suggest that this technique has an incremental prognostic value for mortality in a composite cohort of patients with STEMI and NSTEMI (17). With the current study we can also demonstrate the importance of the GLS for outcome prediction in patients with NSTEMI. Due to the excellent intra- and inter-observer reproducibility (47) and its high sensitivity and specificity to predict the development of MACE (48) myocardial strain analysis by CMR-FT could be used as a robust tool to evaluate clinical endpoints in patients with NSTEMI. When choosing echocardiography to assess LV function after myocardial infarction, it has to be taken into account that this method has a lower detection sensitivity for segmental wall motion abnormalities and underestimate measures of volumes and LV ejection fraction when comparing it to CMR-FT (49). Therefore, CMR-FT derived GLS should be preferred to improve risk stratification in NSTEMI patients.

### Limitations

Some limitations must be considered when interpreting the data. Overall, due to the small sample size of patients ≥80 years, it must be assumed that the study is underpowered. In addition, the stepwise multiple logistic regression revealed no effect of age on the MACE rate conditional on (or given) some more relevant predictors. That means that the three significant predictors explain so much variation in the outcome that age has no additional impact. This result has to be considered with caution because of the resulting reduction of the data base due to missing values in the eligible predictors. Therefore, despite our well-defined patient cohort derived from multicenter randomized data, the results should be confirmed in further research. A higher amount of elderly patients may have led to significant differences in the baseline characteristic, which in turn may influences the results of the logistic regression analysis. Different MRI providers in the seven centers limit the results of this study, but all centers have a high level of expertise in performing CMR examinations and all sites employed the same imaging protocol. Moreover, all data were analyzed in experienced core-laboratories. Additionally, there is also a possible selection bias because of different times of the MRI examination between days 1 to 10 after NSTEMI. Due to comorbidities, unknown reasons or death early after enrollment, 15.2% of all patients in the original trial potentially underwent CMR later or not at all (19). But these limitation gets attenuated by the fact that there is little known about the optimal time-point for CMR and, therefore, further studies are necessary to address this issue. Moreover, due to the small number of clinical events, regression analyses could not be performed separately by subgroup. Nevertheless, in order to appreciate the chosen age category and on the other hand to take age into account in the regression, we decided to evaluate age as a binary variable in addition to the continuous analysis of age. Furthermore, we decided to assess the advisability of CMR-FT for the analysis of MACE according to NSTEMI and to apply this modern method for the consideration of the total cohort. Nevertheless, studies including a higher number of octogenarians and numerous MACE should consider this subject. With the current study, we can make a significant contribution to the field of imaging, especially in elderly patients. The MRI protocols used in this trial provide a relevant amount of information in the context of cardiac MRI diagnostics. Additional information, such as diastolic dysfunction parameters, were not collected and should be included in further studies.

## Conclusion

In this prospective, multicenter analysis, no differences in structural and functional myocardial injury measured by CMR imaging could be observed in octogenarians and non-octogenarians with NSTEMI. Furthermore, our data had not shown that age was independently associated with 1-year clinical outcome despite an increased event rate in octogenarians. Aldosterone antagonist treatment, number of diseased vessels and GLS proved to be independent predictors of MACE within the first year after NSTEMI. CMR-FT was superior to LV EF in predicting MACE at 1-year follow-up.

## Data Availability

The raw data supporting the conclusions of this article will be made available by the authors upon reasonable request.
